# Calcitriol in Sepsis—A Single-Centre Randomised Control Trial

**DOI:** 10.3390/jcm13133823

**Published:** 2024-06-29

**Authors:** Siddhant Jeevan Thampi, Aneesh Basheer, Kurien Thomas

**Affiliations:** 1 Department of Acute Medicine, Sheffield Teaching Hospitals NHS Trust, Sheffield S5 7AU, UK; 2 Department of Internal Medicine, Dr. Moopen’s Medical College, Wayanad 673577, India; basheeraneesh@gmail.com; 3 Department of Internal Medicine, Pondicherry Institute of Medical Sciences, Puducherry 605014, India; kurien123@gmail.com

**Keywords:** calcitriol, sepsis, APACHE II

## Abstract

**Background/Objectives**: Sepsis is a life-threatening organ dysfunction caused by a dysregulated host response to infection. Sepsis is a significant cause of hospital admission and the leading reason for admission to the ICU and is associated with high mortality. Vitamin D has shown promising immunomodulatory effects by upregulating the antimicrobial peptide, cathelicidin. However, previous studies analysing the use of calcitriol in sepsis have shown variable results and did not utilise APACHE II (Acute Physiology and Chronic Health Evaluation II) scores as endpoints. This study evaluates the efficacy of intramuscular calcitriol in patients admitted to the ICU with sepsis, focusing on its impact on APACHE II scores. The primary aim was to determine if intramuscular calcitriol improved APACHE II scores from day 1 to day 7 or discharge from the ICU, whichever was earlier. Secondary outcomes included 28-day mortality, ventilator days, vasopressor days, ICU stay length, adverse events, and hospital-acquired infections in ICU patients. **Methods**: This was a triple-blinded phase III randomised control trial. A total of 152 patients with suspected sepsis were block-randomised to receive either intramuscular calcitriol (300,000 IU) (*n* = 76) or a placebo (*n* = 76). The trial was registered with the Clinical Trials Registry—India (CTRI No: CTRI 2019/01/17066) following ethics committee approval and was not funded. **Results**: There was no significant difference in APACHE II scores between the calcitriol and placebo groups from day 1 to day 7 (*p* = 0.382). There were no significant changes in 28-day mortality (14.4% vs. 17%, *p* = 0.65), number of days on a ventilator (5 vs. 5, *p* = 0.84), number of days on vasopressors (3 vs. 3, *p* = 0.98), length of ICU stay (10 days vs. 11 days, *p* = 0.78), adverse events (27.6% vs. 19.7%, *p* = 0.25), and hospital-acquired infections (17.1% vs. 15.8%, *p* = 0.82). **Conclusions**: There was no effect of intramuscular calcitriol in patients admitted to the ICU with sepsis.

## 1. Introduction

Sepsis is a life-threatening organ dysfunction caused by a dysregulated host response to infection. The balance between pro-inflammatory and anti-inflammatory cytokines is dysregulated in sepsis, with increased levels of IL-6 and 10 and TNF alpha [1,2] along with an imbalance of reactive oxygen species [3]. In sepsis, stimulation of Toll-like receptors [TLRs] causes conversion of 25[OH]D to 1,25[OH]2-D. After 1α-hydroxylation, 25[OH]D enters the monocyte and converts into 1,25[OH]2-D in the mitochondria. It binds to the vitamin D receptor, ultimately acting as a transcription factor for human cathelicidin—an antimicrobial peptide [4]. This forms the principle for which calcitriol has been evaluated for sepsis.

Sepsis is a significant cause of hospital admission and the leading reason for admission to the ICU [5]. A retrospective analysis showed a global incidence of 437 per 100,000 person-years from 1995–2015 [6]. About 12% of admissions into the ICU are due to sepsis [7], associated with a mortality of 34.6%.

Studies appraising the role of vitamin D in critical illness are emerging [8]. However, there is a lot of variability in the evidence. Many studies postulate vitamin D deficiency as a risk factor for sepsis, but these studies were retrospective observational studies using varied definitions of sepsis [9,10]. Some prospective studies [11,12] showed that vitamin D deficiency is seen in 30–75% of critically ill patients but did not affect the outcome of patients in terms of 30-day mortality. There was no difference in mortality between those with vitamin D deficiency and those without [12]. The association between treatment assignment and mortality was not affected by the extent of vitamin D deficiency at baseline.

There is variability in the route of administration even among the randomised control trials. The trial by Leaf et al. [13] used parenteral vitamin D, but it was a small trial involving 67 patients with sepsis following calcitriol, and there was no difference in cathelicidin levels. Ginde et al. [14] used enteral vitamin D in a heterogeneous group of critically ill ICU patients. However, absorption may have been altered in these patients, and this study showed no significant differences in outcomes between intervention and placebo groups.

Moreover, none of the above trials used estimates of APACHE II scores as endpoints. Due to the variability in evidence, our study determined the efficacy of intramuscular calcitriol in sepsis patients admitted to the ICU.

## 2. Materials and Methods

Primary objective: To determine if there was a significant difference in APACHE II scores between calcitriol and placebo groups from day 1 to day 7, or discharge from ICU, whichever was earlier.

Secondary objective: To determine if there was a significant change in 28-day mortality, number of days on ventilator, number of days on vasopressors, length of ICU stay, incidence of adverse events, and hospital-acquired infections in the ICU between calcitriol and placebo groups.

Patients: The trial was a single-centre, triple-blinded randomised controlled phase 3 superiority trial with a 1:1 allocation ratio conducted from January 2019 to August 2020 in non-COVID patients. Patients recruited were 18 years of age or older. Only non-COVID patients were recruited, as the trial had already begun prior to the COVID pandemic.

Inclusion criteria: Patients with suspected sepsis (either presumed or confirmed to be due to an infectious etiology) as defined by the presence of SIRS ≥ 2 and qSOFA ≥ 2 were included in the study.

Exclusion criteria: Pregnancy, brain injury after cardiopulmonary arrest before recruitment, patients who were severely ill with APACHE II > 45 (85% mortality) and unlikely to survive for more than 24 h, baseline hypercalcemia > 10.8 mg/dL, history of therapy with high-dose vitamin D3 in the past six months, patients with platelets < 50,000 or INR > 1.3, and patients with chronic kidney disease who had received any form of vitamin D supplementation were excluded.

**Figure 1:**
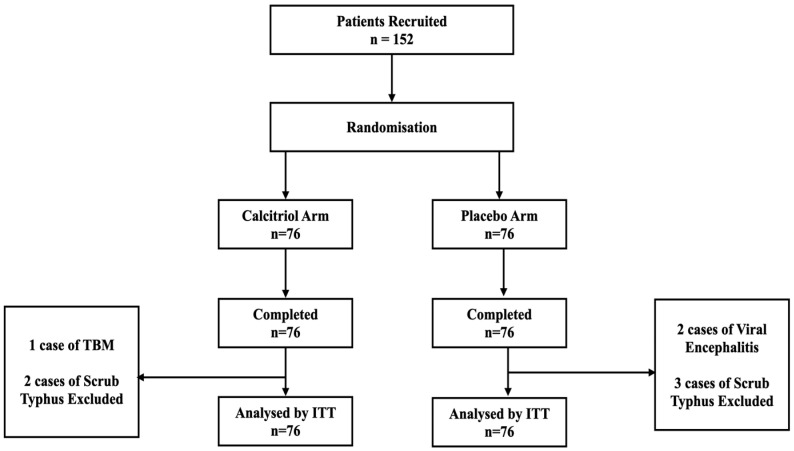
CONSORT flow diagram (ITT—intention to treat; TBM—tubercular meningitis).

Sample size: An *a priori* power analysis was conducted using G*Power to determine the minimum sample size required to test the study hypothesis. Results indicated the required sample size to achieve 80% power for detecting a medium effect (Partial η2 = 0.10) at a significance criterion of α = 0.05 was 124 for a two-way repeated measures ANOVA (within-subject factors of APACHE II score and between-subject factors—calcitriol vs. placebo). With a presumptive loss to follow-up of 10%, the final sample size required was calculated to be 139. We decided to recruit 150 patients for the study. Since it was a block-randomisation of 4 samples per block, there were 38 blocks of 4. Hence, 152 participants were recruited for the study (Figure 1).

Randomisation: An independent statistician generated a computer-generated sequence of random numbers. The primary investigator enrolled patients meeting the criteria. Allocation was concealed using sealed envelopes. An independent person assigned participants to the intervention using these sealed envelopes and had no other role in the study.

Recruited patients were randomised within the first six hours of ICU admission into two groups, receiving either calcitriol 300,000 IU intramuscularly as a single dose or a placebo. The placebo was an inert solution (sterile water for injection), similar in colour and quantity to the intervention drug.

Patients and the primary investigator were blinded to the intervention. A participant information sheet and consent form were provided to the participants. If they agreed to participate in the study, the consent form was signed and obtained. Participants were free to withdraw consent at any point in time. If the patient was unconscious or had poor sensorium, then the study details were provided to the next of kin, and the primary investigator obtained consent from the next of kin. All patients were followed up for 28 days.

Statistics and data analysis: IBM SPSS version 27 was used for the analysis of the data. Intention-to-treat analysis was performed for primary and secondary outcomes. No interim analyses were performed.

Descriptive statistics were used to present the clinical profile of patients. Continuous data were represented as means and standard deviation, and categorical data as proportions with confidence intervals. Two groups were compared using the chi-square test for categorical variables. CONSORT reporting guidelines were followed. A linear mixed model with two-way repeated measures ANOVA (analysis of variance) was performed to determine the following:

1. If there was any significant change in serial APACHE II scores across seven days (within-subject factors—APACHE score trend from day 1–7).
2. If there was any difference in change of APACHE II scores between the groups of placebo and calcitriol (between-subject factors—calcitriol vs. placebo).
3. If there was a significant interaction (time*treatment interaction) among the within-subject factors (APACHE score) and the between-subject factors (calcitriol vs. placebo).

All were represented as *p* values and <0.05 was considered as statistically significant.

Ethical considerations: Data confidentiality was maintained, and each patient was assigned a unique serial number to maintain confidentiality. Individual identifying data were removed in any publication of this data.

The study protocol was presented to the institute research and ethics committee, and approval for the conduct of the study was obtained (RC 18/74). This clinical trial was registered with the Clinical Trials Registry of India (CTRI) after obtaining approval from the institute’s ethics committee (No: CTRI 2019/01/17066).

Quality control of data was ensured using a preformed proforma, double data entry into software, and random quality control checks for every 10% of samples. Data collection was performed by the principal investigator in preformed proforma and entered in MS Excel. The trial was not funded.

## 3. Results

### 3.1. Baseline Characteristics

The baseline characteristics are described below (Table 1). Characteristics were similar in both groups.

### 3.2. Primary End Point

**Figure 2:**
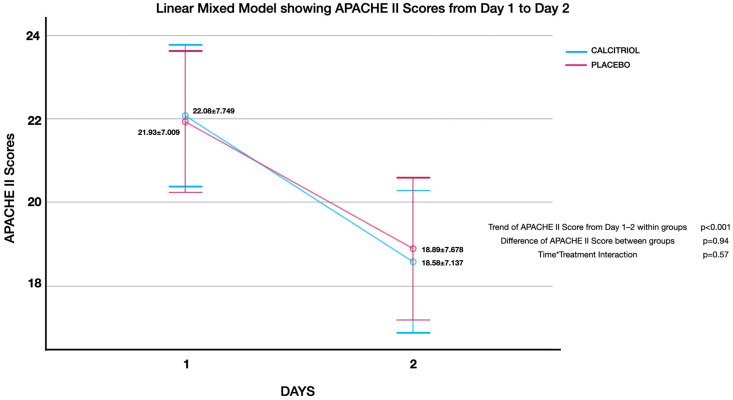
APACHE II scores from day 1 to day 2: From day 1 to day 2, APACHE II scores showed an improvement within both groups (*p* < 0.001), but there was no significant difference between groups (*p* = 0.94). There was no interaction between the number of days and APACHE II scores (time*treatment interaction, *p* = 0.57), suggesting that the treatment does not differentially affect the rate of improvement in APACHE II scores compared to the placebo and standard care.

**Figure 3:**
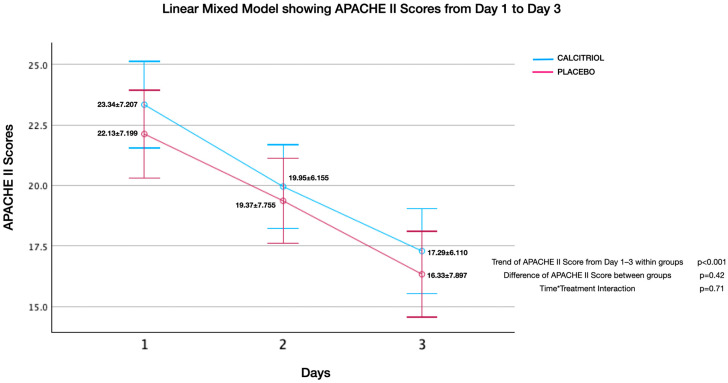
APACHE II scores from day 1 to day 3: APACHE II scores showed an improvement within both groups (*p* < 0.001), but there was no significant difference between groups (*p* = 0.42). There was no interaction between the number of days and APACHE II scores (time*treatment interaction, *p* = 0.71), suggesting that the treatment does not differentially affect the rate of improvement in APACHE II scores compared to the placebo and standard care.

**Figure 4:**
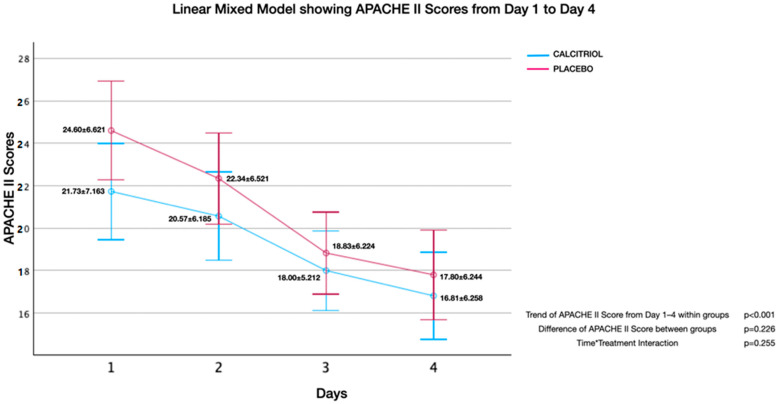
APACHE II scores from day 1 to day 4: APACHE II scores showed an improvement within both groups (*p* < 0.001), but there was no significant difference between groups (*p* = 0.22). There was no interaction between the number of days and APACHE II scores (time*treatment interaction, *p* = 0.26), suggesting that the treatment does not differentially affect the rate of improvement in APACHE II scores compared to the placebo and standard care.

**Figure 5:**
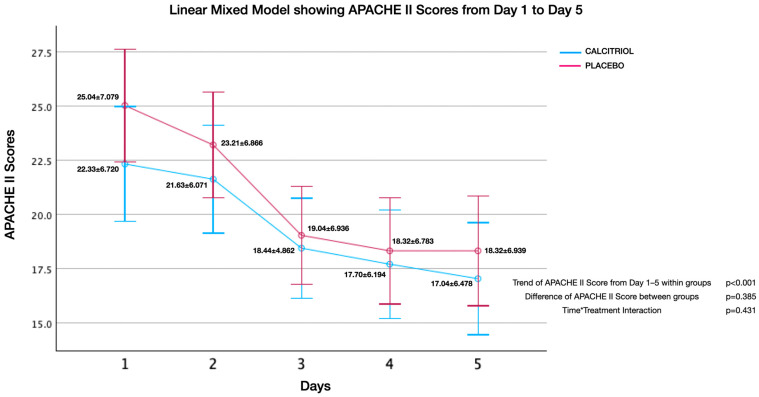
APACHE II scores from day 1 to day 5: APACHE II scores showed an improvement within both groups (*p* < 0.001), but there was no significant difference between groups (*p* = 0.39). There was no interaction between the number of days and APACHE II scores (time*treatment interaction, *p* = 0.43), suggesting that the treatment does not differentially affect the rate of improvement in APACHE II scores compared to the placebo and standard care.

**Figure 6:**
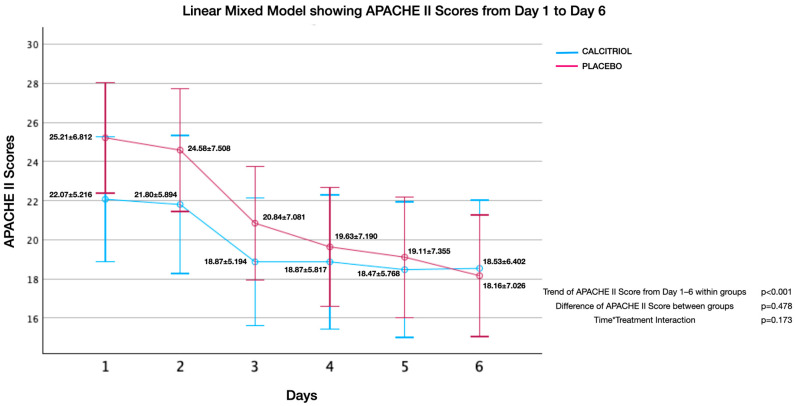
APACHE II scores from day 1 to day 6: APACHE II scores showed an improvement within both groups (*p* < 0.001), but there was no significant difference between groups (*p* = 0.48). There was no interaction between the number of days and APACHE II scores (time*treatment interaction, *p* = 0.17) suggesting that the treatment does not differentially affect the rate of improvement in APACHE II scores compared to the placebo and standard care.

**Figure 7:**
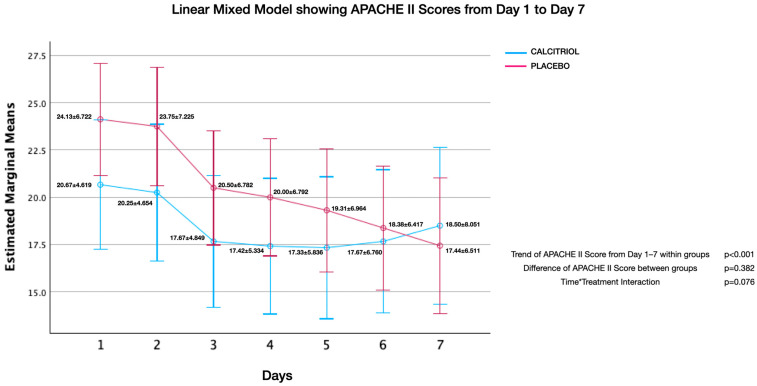
APACHE II scores from day 1 to day 7: APACHE II scores showed an improvement within both groups (*p* < 0.001), but there was no significant difference between groups (*p* = 0.38). There was no interaction between the number of days and APACHE II scores (time*treatment interaction, *p* = 0.08), suggesting that the treatment does not differentially affect the rate of improvement in APACHE II scores compared to the placebo and standard care.

Effect of calcitriol versus placebo on APACHE II score (Figure 2, Figure 3, Figure 4, Figure 5, Figure 6 and Figure 7):

Although both groups had significant reduction in APACHE II Scores over time, there was no significant difference of APACHE II Scores between groups. There was no interaction between the number of days and APACHE II scores (time*treatment interaction, suggesting that the treatment does not differentially affect the rate of improvement in APACHE II scores compared to the placebo and standard care. This finding was uniformly noted from Day 1 to Day 2 upto Day 7 (Figure 2, Figure 3, Figure 4, Figure 5, Figure 6 and Figure 7). The individual subsets are listed below.

**Figure 8:**
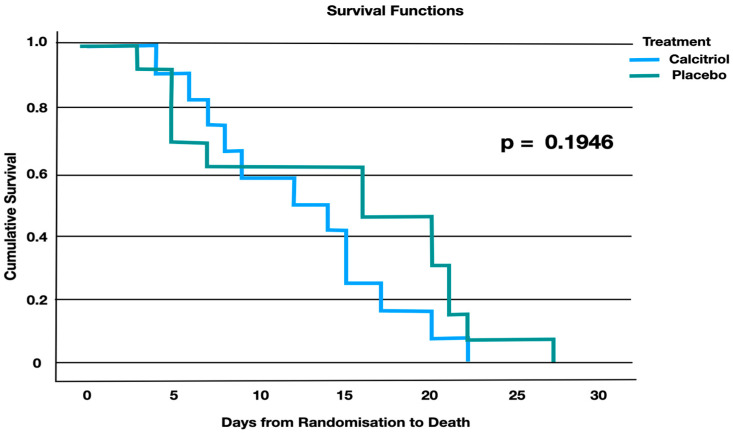
Kaplan–Meier survival analysis. There was no significant difference in mortality in both groups at 28 days.

There was no significant difference in mortality in both groups at 28 days (Table 2, Figure 8).

### 3.3. Primary End Point

There was no benefit in mortality, lengths of hospital and ICU stay, duration of mechanical ventilation, duration of vasopressors, and incidence of new-onset hospital-acquired infections and adverse events (Table 3).

## 4. Discussion

Our study on the role of intramuscular calcitriol did not show significant difference in the APACHE II score between those who received vitamin D and those who received the placebo. This is discussed below.

Several significant differences exist between the previous trials evaluating the role of vitamin D levels in sepsis. Some studies show that vitamin D levels may fail to predict sepsis [15]. At the same time, other studies show that vitamin D deficiency is a risk factor for developing sepsis [8,9,16,17]. Even so, vitamin D deficiency may not influence prognosis and mortality within the intensive care unit [11,12,15].

In a double-blind, randomised, placebo-controlled study among 67 critically ill patients by Leaf et al. [13], the measured outcomes were plasma cathelicidin levels. The study was a double-blinded RCT. The patients were randomised to receive a single dose of calcitriol 2 mg intravenously versus a placebo. The primary outcome measured was plasma cathelicidin protein levels assessed 24 h after the study drug administration. Secondary outcomes included leukocyte cathelicidin mRNA expression, plasma cytokine levels (IL-10, IL-6, tumour necrosis factor-a, IL-1b, and IL-2), and urinary kidney injury markers. Patients randomised to calcitriol (*n* = 36) versus placebo (*n* = 31) had similar plasma cathelicidin protein levels at 24 h (*p* = 0.16). Calcitriol administration did not increase plasma cathelicidin protein levels in critically ill patients with sepsis. There was no difference in clinical outcomes; however, the study was not powered enough to detect a change.

A Chinese study by Ding et al. [18] in 2017 randomised 57 patients with sepsis by the 2012 definition [19]. Twenty healthy volunteers were also recruited, and their vitamin D levels were measured. The levels of serum 25(OH)D3 in the sepsis group and SIRS group were significantly lower than those in the healthy control group.

The study also divided these 57 patients into general sepsis (*n* = 15) and severe sepsis (*n* = 27). The sepsis patients who had a deficiency were divided into treatment with calcitriol supplemented with 300,000 IU vitamin D3 and a placebo (injected 1 mL physiological saline). However, the study did not mention how many had a deficiency. The study did not mention whether patients with septic shock had a lower vitamin D level.

The 28th day was set as the endpoint, and the patients with sepsis were divided into a survival group and a mortality group. The levels of calcitriol in the sepsis group and SIRS group were significantly lower than those in the healthy control group. In patients with sepsis, there was no significant difference in the duration of mechanical ventilation [hours: 41.00 (7.50, 82.50) vs. 67.00 (4.75, 127.75)], length of ICU stay (days: 5.48 ± 4.08 vs. 6.68 ± 4.87) and 28-day mortality (10.34% vs. 17.86%) between the D3 treatment group and the placebo group. The difference in our study is that we used APACHE II scores as an outcome, while the study by Ding et al. evaluated it as a risk factor. We did not measure vitamin D in our study due to vitamin D deficiency failing to predict sepsis [13,14].

In 2014, Amrein et al. [20] showed that in the VITdAL-ICU study, which involved 475 critically ill patients (237 in the vitamin D3 group and 238 in the placebo group), vitamin D3 vs. placebo was given enterally once at a dose of 540,000 IU followed by monthly maintenance doses of 90,000 IU for five months. There was no significant difference in duration of hospital stay, in-hospital mortality, and six-month mortality between the two groups. However, enteral vitamin D may have an impeded absorption within the gut.

Ginde et al. [14], in 2019, conducted a randomised, double-blind, placebo-controlled, phase 3 trial of early vitamin D supplementation in critically ill, vitamin D-deficient patients at high risk for death. Randomisation was performed within 12 h of admission into the ICU. Patients received a single dose (enteral) of 540,000 IU of calcitriol or a matched placebo. The primary endpoint was 90-day all-cause, all-location mortality. The mortality at 90 days was 23.5% in the calcitriol group (125 of 531 patients) and 20.6% in the group of patients who received a placebo (109 of 528 patients). The difference was 2.9% points; 95% CI, −2.1 to 7.9; *p* = 0.26.

There were no differences between groups regarding secondary clinical, physiological, or safety endpoints. The extent of vitamin D deficiency at baseline did not affect the association between the treatment assignment and mortality.

Only a few randomised control trials were conducted assessing the effect of vitamin D on sepsis. Further studies done by Bjelakovic [21] in a meta-analysis of 94,148 patients showed a relative risk reduction in mortality by 6% (RR 0.94, 95% CI 0.91 to 0.98, I^2^ = 0%), and two good quality RCTs done by Amrein [20] and Ginde [14] showed no benefit of vitamin D in terms of mortality and hospital stay. However, unlike our study, the Amrein and Ginde trials evaluated the use of vitamin D given enterally and not parenterally.

The strengths of our trial are that there was little loss to follow-up, and those lost to follow-up were analysed by ITT. The trial is generalisable, and all relevant outcomes were measured. The limitation of the trial was that randomisation was not stratified by the etiology of sepsis in these patients.

Due to the heterogeneity of evidence of vitamin D levels in predicting sepsis and its outcomes and the cost of testing, we did not measure vitamin D levels. There were logistic difficulties in evaluating the levels of cathelicidin and other biomarkers. We did not evaluate the impact on COVID-19 patients, as the trial commenced in January 2019. The qSOFA and SIRS score was used for ease of recruitability and rapid intervention with vitamin D. However, the SOFA score may generate a detailed profile of these patients within the ICU. The trial was too small to obtain a significant effect in patients admitted with septic shock.

## 5. Conclusions

In conclusion, this study indicates that intramuscular calcitriol was not better than a placebo in improving outcomes of patients with sepsis admitted to the hospital. Future trials must identify if calcitriol has any role in specific subgroups of sepsis, with a focus on particular sources, septic shock, or COVID patients. It is possible that upregulation of cathelicidins can be dose-dependent, and it would be useful to evaluate the role of high dose vitamin D (600,000 IU) in sepsis.

## Figures and Tables

**Table 1:** Baseline Characteristics.

		Calcitriol (*n* = 76)	Placebo (*n* = 76)	Overall *n* = 152
Age (Years)		61.68 ± 14.08	60.78 ± 13.83	61.2 ± 13.90
Males		47/76 (61.8%)	48/76 (63.1%)	95/152 (62.5%)
Females		29/76 (38.2%)	28/76 (36.8%)	57/152 (37.5%)
qSOFA	Resp Rate > 22	70/76 (92.1%)	69/76 (90.7%)	139/152 (91.4%)
	Altered Sensorium GCS < 15	54/76 (71.0%)	67/76 (88.1%)	121/152 (79.6%)
	Systolic BP < 100	37/76 (48.6%)	25/76 (32.8%)	62/152 (40.7%)
SIRS	Temp < 36 or > 38	41/76 (53.9%)	37/76 (48.6%)	78/152 (51.3%)
	HR > 90	51/76 (67.1%)	50/76 (65.7%)	101/152 (66.4%)
	Tachypnea > 20	70/76 (92.1%)	69/76 (90.7%)	139/152 (91.4%)
	WBC < 4000 or > 12,000	55/76 (72.3%)	54/76 (71.0%)	109/152 (71.7%)
Focus of Sepsis	Urosepsis	22/76 (28.9%)	29/76 (38.1%)	51/152 (33.5%)
	Undifferentiated	25/76 (32.8%)	16/76 (21.0%)	41/152 (26.9%)
	Pulmonary	10/76 (13.1%)	16/76 (21.0%)	26/152 (17.1%)
	Wound	8/76 (10.5%)	10/76 (13.1%)	18/152 (11.8%)
	Skin	2/76 (2.6%)	5/76 (6.5%)	7/152 (4.6%)
	Central Nervous System	3/76 (3.9%)	4/76 (5.2%)	7/152 (4.6%)
	Central Line	4/76 (5.2%)	1/76 (1.3%)	5/152 (3.2%)
Baseline APACHE II Score		22.42 ± 7.55	22.28 ± 7.05	22.35 ± 7.28
Comorbidities	Diabetes	45/76 (59.2%)	44/76 (57.8%)	89/152 (58.5%)
	Hypertension	33/76 (43.4%)	34/76 (44.7)	67/152 (44.0%)
	Ischemic Heart Disease	14/76 (18.4)	12/76 (15.7)	26/152 (17.1%)

Table 1—Description of baseline characteristics of analysed participants.

**Table 2:** Effect of calcitriol versus placebo on 28-day mortality.

Treatment	Total Number of Deaths	Total Patients	Percentage
Calcitriol	11	76	14.4%
Placebo	13	76	17.1%
Overall	24	152	*p* = 0.65 RR 0.84 (0.40–1.76)

**Table 3:** Secondary outcomes.

		Calcitriol (*n* = 76)	Placebo (*n* = 76)	Overall *n* = 152	*p*-Value	dF	RR
Mechanical Ventilation							
Number of patients		14/76 (18.4%)	15/76 (19.7%)	29/152 (19%)	0.83	1	0.93 (0.48–1.79)
Median Duration		5 days (IQR 9 days)	5 days (IQR 12 days)		0.84		
Vasopressors							
Single	Noradrenaline	14/76 (18.4%)	15/76 (19.7%)	29/152 (19%)	0.83	1	0.93 (0.48–1.79)
Double	Noradrenaline + Dobutamine	1/76	0/76	1/76	0.31	1	
	Noradrenaline + Dopamine	4/76	4/76	8/76	0.99	1	
	Noradrenaline + Vasopressin	2/76	2/76	4/76	0.99	1	
Triple	Noradrenaline + Dobutamine + Vasopressin	1/76	1/76	2/76	0.99	1	
Quadruple		1/76	0/76	1/76	0.31	1	
Total		23/76 (30.2%)	22/76 (28.9%)	45/152 (29.6%)	0.86	1	1.05 (0.64–1.70)
Median Duration		3 days (IQR 4.25 days)	3 days (IQR 4 days)		0.98		
ICU Stay							
ICU Stay	Median Duration	3 days (IQR 2 days)	3 days (IQR 2.5 days)		0.78		
Hospital Stay							
Hospital Stay	Median Duration	10 days (IQR 8.5 days)	11 days (IQR 11.5 days)		0.50		
Hospital Acquired Infections							
New Onset UTI		5/76 (6.5%)	5/76 (6.5%)	10/152 (6.5%)	0.99	1	1.00 (0.30–3.31)
New Onset VAP		6/76 (7.8%)	4/76 (5.2%)	10/152 (6.5%)	0.51	1	1.5 (0.44–5.10)
New Onset Central Line Sepsis		2/76 (2.6%)	3/76 (3.9%)	5/152 (3.2%)	0.64	1	0.66 (0.11–3.87)
Total		13/76 (17.1%)	12/76 (15.8%)	25/76 (32.9%)	0.82	1	(0.44–2.45)
Adverse Events							
Need for Haemodialysis		16/76 (21.0%)	10/76 (13.1%)	26/152 (17.1%)	0.19	1	1.6 (0.77–3.29)
Seizures		3/76 (3.9%)	3/76 (3.9%)	6/152 (3.9%)	0.99	1	1.00 (0.20–4.80)
Feeding Intolerance		2/76 (2.7%)	2/76 (2.8%)	4/152 (2.7%)	0.97	1	1.00 (0.14–6.91)
Total		21/76 (27.6%)	15/76 (19.7%)	36/152 (23.6%)	0.25	1	1.00 (0.56–2.53)

dF—Degrees of freedom; RR—Relative risk; IQR—Inter-quartile range; UTI—Urinary tract infection; VAP—Ventilator-associated pneumonia.

## Data Availability

The Master Data Sheet has been uploaded as part of the Supplementary Materials Table S1: Data Sheet.

## Supplementary Materials

The following supporting information can be downloaded at: https://www.mdpi.com/article/10.3390/jcm13133823/s1, Table S1: Data Sheet.
